# A comparison of socioeconomic status and mental health among inner-city Aboriginal and non-Aboriginal women

**DOI:** 10.1080/22423982.2017.1340693

**Published:** 2017-07-26

**Authors:** Kevin Hamdullahpur, Kahá:wi J. Jacobs, Kathryn J. Gill

**Affiliations:** ^a^ McGill University Health Centre, Montréal, Quebec, Canada; ^b^ Psychiatry Department, McGill University, Montreal, Quebec, Canada

**Keywords:** Violence, women, First Nations, Aboriginals, physical and sexual abuse, mental health

## Abstract

Aboriginal women in urban areas have been reported to experience high rates of poverty, homelessness, interpersonal violence, and health problems. However, there are few prior ethnocultural comparisons of urban women from similar socioeconomic backgrounds. The current study explored the mental and physical health of Aboriginal and non-Aboriginal women accessing social services agencies and shelters.

Half of the sample (n=172) was Aboriginal (48.3%). The lifetime rate of physical abuse was significantly higher in Aboriginal women, and they were more likely to have been victims of violence or crime in the past year (A=50.6%, NA=35.6%, p<0.05). Rates of teenage pregnancy (<18 years of age) were significantly higher among Aboriginals (A=51.3%, NA=30.6%, p<0.05) and they reported more parental drug/alcohol problems (A=79.2%, NA=56.5%, p<0.05). Aboriginal women were also more likely to have previously received treatment for a drug or alcohol problem. There were no differences in self-reported physical health, medication use, hospitalisations, and current substance misuse. Irrespective of ethnicity, lifetime rates of anxiety, depression and suicide attempts were extremely high. Future research should explore the effects of individual resources (e.g. social support, family relations) and cultural beliefs on women’s ability to cope with the stress of living with adverse events, particularly among low SES women with children.

## Introduction

The social marginalisation of Aboriginal women in urban centres is a growing Canadian public health issue. According to the 2011 National Household Survey (NHS) 56% of Aboriginal Canadians live in urban centres [1], and several studies have shown that compared with men, a disproportionately high number of Aboriginal women have moved into urban areas [2,3]. Movement from reserve to city is often motivated by a number of factors relating to home (e.g. lack of adequate housing, isolation) and community (e.g. lack of educational and employment opportunities, low standard of living) [4].

Numerous reports have underscored the socioeconomic disadvantages faced by many Aboriginal Canadians living in both reserves and large cities [5–8]. Canadian census data revealed the median income of Aboriginal people in 2006 was $18,962, markedly lower than the income of non-Aboriginal people ($27,097) [9]. More recently, a study of 554 First Nations adults in Hamilton found that 78% earned less than $20,000 per year [8]. Low socioeconomic status (SES) is often associated with unhealthy behaviours (inactivity, poor nutrition), substance abuse and poor social support [10–14]. A large body of evidence has also shown a relationship between SES and rates of abuse [15,16].

Violence has been emphasised as a major issue among Aboriginal groups [17–20]. For example, in 2009 the General Wellness Survey found that 67,000 Aboriginal women were victims of violence within the past year, nearly 3 times the rate of non-Aboriginal women [21]. In general it appears that Aboriginal women experience high levels of severe physical abuse and sexual assault [22], and are at significantly greater risk of experiencing violence from a former partner following separation [23]. Strikingly, Canadian Aboriginal women are 8 times more likely to be homicide victims compared with non-Aboriginals [24].

The literature suggests that low SES and the experience of violence and sexual abuse foster poor physical and mental health [25–29]. In a study of urban Aboriginals, Jacobs and Gill [2] found that those who had experienced physical/emotional abuse were more likely to have substance abuse and emotional distress (depression, suicide attempts). Studies among First Nations groups have also demonstrated links between lifetime abuse and elevated rates of depression and attempted suicide [30,31]. This association is not limited to Aboriginal women, however; a recent US study of 1073 low SES women found a relationship between childhood abuse (physical, emotional or sexual) and current anxiety disorders, posttraumatic stress disorder, bipolar disorder, physical health problems and domestic violence [28]. Therefore, the current study was designed to compare and contrast abuse history, physical health and mental health status among low-income Aboriginal and non-Aboriginal women in one of Canada’s largest metropolitan areas. The study sample consisted of socioeconomically disadvantaged women seeking help with obtaining basic services including food and shelter. Aboriginal and non-Aboriginal women were compared on demographic characteristics, family and social relationships, medical status, mental health and drug/alcohol abuse.

## Methods

### Participants and research sites

Eligible participants were help-seeking Aboriginal and non-Aboriginal women who were at least 16 years of age, that were accessing services at 1 of 8 sites including drop-in centres, social service agencies, and shelter organisations in the Montreal region. The word Aboriginal used in this study refers to a heterogenous ethnocultural group from a particular geographic area, who are differentiated linguistically, culturally, historically and politically. This population includes status and non-status Native (including Cree, Mohawk, Innu, Mi’kmaq, Attikamekw, Naskapi, Ojibway, Malecite and Algonquin), Inuit and Métis people.

Each recruitment site provided a wide range of basic services including information on how to access medical services, housing, food and social activities. Shelter services also offered some individual or group counselling, assistance in applying for financial aid, family allowance or low-cost housing, and help obtaining identification such as social insurance cards, birth certificates and medicare cards. Two of the sites specifically catered to Aboriginal women, offering help in obtaining Native status cards, and referrals to Native-run drug/alcohol treatment centres. None of the recruitment sites directly provided medical or addiction treatment services. Research assistants made weekly visits to participating organisations where they were introduced by staff to all clients during group meetings at shelters, and in common rooms at the 2 service centres. Bilingual printed notices of the study were also posted. Following informed consent, interviews were conducted in private settings, and respondents were remunerated for their participation with $20.00 in gift certificates.

### Interviews and study instruments

#### Substance abuse and psychological problems

Information on substance use and psychopathology (e.g. depression, anxiety, suicidal ideation, etc.) was collected using the Addiction Severity Index (ASI) in French (RISQ, 1996) and English [32]. Interviews were conducted in the subject’s preferred language, with 1 of 4 female interviewers (2 Aboriginal, 2 non-Aboriginal). The ASI is a validated, semi-structured interview designed to collect information in 7 domains including sociodemographics, medical status, employment and education, legal status, and recent and lifetime physical and sexual abuse. The medical status section of the ASI was supplemented with additional questions regarding pregnancy (number of pregnancies in lifetime, teenaged pregnancies). Within each domain, respondents identified the number, duration, frequency and intensity of symptoms experienced during the past 30 days. Items relating to recent problem severity are weighted to create a composite severity score. Composite scores range from 0 (no significant problem) to 1 (extreme problem). The drug and alcohol subscales have been shown to have interrater reliability ranging from 0.86–0.96 and test–retest reliabilities of 0.92 [33]. The ASI has been widely employed in Quebec, and had been recommended by the Comité-Conjoint MSSS-Réseau sur la sélection d’instruments d’évaluation de la clientele [The Provincial Joint Committee of the Health and Social Services Ministry and the Network on the Selection of Patient Evaluation Instruments] [34].

#### Psychological distress

The Beck Depression Inventory (BDI) is a 21-item self-report that rates the severity of cognitive, affective, somatic, and vegetative symptoms of depression on a 4-point scale from neutral to severe. The total score can range from 0 to 63, reflecting the overall level of depression experienced in the week prior to the test [35]. The psychometric properties of the BDI are good, with internal consistencies ranging from 0.86 (in clinical samples) and 0.81 (in non-clinical samples) [36]. Bilingual versions of this instrument were used, in French [37] and English [35].

#### Childhood physical/sexual abuse and neglect

Additional information on childhood physical, sexual and emotional abuse was collected using French and English versions of the Child Abuse and Trauma Scale (CATS). The CATS is a 38-item self-report questionnaire created to measure the existence of childhood abuse in a manner sensitive to individuals’ perceptions of the impact of childhood abuse [38]. Each item is rated on a 5-point scale (0–4) yielding an overall index of childhood trauma as well as 3 subscales of negative home environment/neglect, sexual abuse, and punishment. The CATS subscales have been shown to have internal consistency ranging from 0.63–0.86. Test–retest reliabilities were 0.91 for the neglect/negative home atmosphere subscale, 0.85 for the sexual abuse subscale, and 0.71 for the punishment subscale [38].

### Data analysis

Microsoft Excel was used to construct a database containing all information collected from the interviews. Data analyses were conducted using SPSS version 22 (IBM Corp). Baseline comparisons between Aboriginal and non-Aboriginal women were conducted using Students *t*-tests and ANOVAs for continuous variables or Chi-square test for categorical variables. Groups were compared in terms of demographic variables, education and employment, abuse history, physical and mental health characteristics. Post-hoc tests were performed using a Bonferroni correction.

## Results

### Demographics

Demographic characteristics of the general sample (n=172) are provided in Table 1. Half of the sample was Aboriginal (48.3%), and the breakdown by nation was Inuit (31.3%), Cree (25.3%), Mohawk (8.4%) and Innu (8.4%). Nearly two-thirds (62.7%) of Aboriginal women reported a Native language as their mother tongue, with the remainder being English (27.7%) or French (9.6%). Among non-Aboriginal women, 91% were Caucasian, with mother tongue reported as 62.9% French, 23.6% English and 13.5% other. Aboriginal women reported having significantly more children [t(162)=5.57, p<0.0001] compared with non-Aboriginal women. Non-Aboriginal women reported living in Montreal significantly longer than Aboriginal women [t(166)=−7.1, p=0.0001], and had significantly more family members living in the city [t(167)=−2.79, p=0.007]. There were no differences between groups with regards to closeness or conflict with family members. In addition, Aboriginal and non-Aboriginal women were similar in terms of age, marital status, legal status and religious preferences.

**Table 1.** Selected demographics stratified by ethnocultural background (n=172).

	Non-Aboriginal (n=89)	Aboriginal (n=83)
**Age (**± **SEM)**	39.9 ± 2.05	37.7 ± 1.46
**Marital status**		
Single	51%	42%
Married/common-law	5%	3%
Separated/divorced	31%	37%
Widowed	2%	1%
**Lived with (past 3 years)**		
Partner/family	39.3%	56.6%
Friends	5.6%	4.8%
Alone	34.8%	20.5%
Nothing stable	18%	14.5%
**Number of children**	1.2	2.7
**Number of dependents (**± **SEM)**	0.3 ± 0.07	0.7 ± 0.2
**Number family members in Montreal** (± SEM) *	3.5 ± 0.56	1.8 ± 0.25
Number years lived in Montreal (±SEM) *	24.6 ± 1.9	7.1 ± 1.4
**Living in a shelter**	66.3%	78.3%
**Why in shelter?**		
Temporary housing	38.6%	48.8%
Fleeing abusive relationship	18.2%	19.5%
Counselling/support/other	9.1%	9.8%

Groups were compared using Student’s t-tests and Chi-square analysis. Values represent the group percentage, or mean ± SD.

* Significant differences between groups p < 0.05, corrected for multiple comparisons.

### Housing and socioeconomic status

Nearly all Aboriginal and non-Aboriginal women in this sample experienced financial and employment difficulties. The majority of both non-Aboriginal and Aboriginal women were residing in shelters at the time of interview. The primary reasons for staying in shelters were a temporary need for housing and fleeing abusive relationships. As shown in Table 2, the majority of women sampled had no post-secondary education, and non-Aboriginal women reported significantly more years of education compared with Aboriginal women [t(169)=−2.99, p=0.003]. The majority of both groups stated welfare was their primary source of income, and reported similar, low past-month earnings. Both groups had identical high ASI employment composite score severity ratings.

**Table 2.** Indicators of socioeconomic status stratified by ethnocultural background (n=172).

	Non-Aboriginal (n=89)	Aboriginal (n=83)
**Number years of education** (± SEM) *	11.8 ± 0.4	10.3 ± 0.3
**Employment pattern (past 3 years)**		
Employed	30.3%	35.4%
Unemployed or student/retired	69.7%	64.6%
**Currently on welfare**	69.7%	61.0%
**Monthly income** (± **SEM)**	$618.10 ± 34.93	$573.57 ± 45.33
**Mean ASI Employment Composite Severity Score (± SEM)†**	0.9 ± 0.2	0.9 ± 0.2

Groups were compared using Student’s *t*-tests and Chi-square analysis. Values represent the group percentage, or mean ± SEM.

* Significant differences between groups p<0.05, corrected for multiple comparisons.

†ASI – Addiction Severity Index composite scores range from 0 to 1.0, with higher scores indicating greater problem severity.

### Childhood & lifetime trauma and adverse events

Adverse childhood and adult events were extremely prevalent among this sample of women, and several differences emerged between Aboriginal and non-Aboriginal women (Table 3). Aboriginal women were significantly more likely to have had a sexual experience with an adult before they were 14 [χ^2^(1)=5.97, p=0.015]. Aboriginal women were also significantly more likely to report a teenage pregnancy [χ^2^(1)=7.16, p=0.007], more problems within their homes, including a higher likelihood of witnessing sexual abuse as children [χ^2^(1)=6.74, p=0.009], and more parental drug/alcohol problems [χ^2^(1)=9.5, p=0.002]. Although lifetime rates of sexual abuse did not vary between groups, Aboriginal women had significantly greater CATS sexual abuse scores compared with non-Aboriginal women [t(110)=2.83, p=0.005], indicating more frequent or more severe sexual abuse experiences in childhood.

**Table 3.** Trauma and adverse life events stratified by ethnocultural background (n=172).

	Non-Aboriginal (n=89)	Aboriginal (n=83)
**Childhood**		
Had sex with an adult before 14*	34.1%	53.4%
Had a teen pregnancy (<18 years of age)*	30.6%	51.3%
Ever witnessed the sexual abuse of another family member*	15.7%	33.3%
**Ever witnessed the physical abuse of another family member**	64.3%	64.0%
**CATS sexual abuse severity score** (± SEM)*	5.17 ± 0.5	7.03 ± 0.8
**Lifetime**		
One or both parents ever had a drug/alcohol problem *	56.5%	79.2%
One or both parents ever had a psychological problem *	63.5%	47.3%
Victim of violence or crime in the past year*	35.6%	50.6%
**Emotional abuse**	85.1%	82.9%
**Sexual abuse**	69.0%	74.4%
Physical abuse*	76.7%	91.5%

Groups were compared using Student’s *t*-tests and Chi-square analysis. Values represent the group percentage, or mean ± SEM.

*** sample for this comparison consisted of women who had a pregnancy lifetime, n=63.

*Significant differences between groups p<0.05, corrected for multiple comparisons.

Lifetime experience of sexual and emotional abuse was pervasive in this sample; however, rates did not differ significantly between groups. Significantly more Aboriginal women reported being the victim of violence or a crime within the past year [χ^2^(1)=3.85, p=0.05). Aboriginal women also reported significantly greater rates of lifetime physical abuse [χ^2^(1)=6.74, p<0.009].

### Physical health

This sample of women experienced a number of medical problems in the past month, and the past year (Table 4). Non-Aboriginal women reported significantly higher rates of fatigue than Aboriginal women [χ^2^(1)=6.78, p=0.009]; however, they did not differ in rates of chronic medical problems, or in the likelihood of seeking care from a medical practitioner for a health problem. Differences in help seeking were apparent; non-Aboriginal women were more likely to have sought care from a general practitioner (GP), and Aboriginal women largely sought care from hospitals or clinics [Aboriginals: GP 25%, hospital 75%; non-Aboriginals: GP 52.6%, hospital 47.4%; χ^2^(1)=9.07, p=0.003].

**Table 4.** Physical health stratified by ethnocultural background (n=172).

	Non-Aboriginal (n=89)	Aboriginal (n=83)
**In the past year experienced**		
Pain in legs, arms, stomach	70.8%	62.2%
Chest pains	34.8%	40.2%
Fatigue*	83.1%	65.9%
Insomnia	68.5%	61.7%
**Has a chronic medical problem**	58.4%	52.4%
**Sought help for medical problems in the past year**	67.1%	74.4%
**# Days medical problems in** **past 30 (± SEM)**	18.0 ± 1.4	15.1 ± 1.5
**Mean ASI Medical Composite Severity Score (± SEM)†**	0.6 ± 0.04	0.5 ± 0.04
**# Times hospitalised in lifetime** **(± SEM)**	4.8 ± 0.8	4.1 ± 0.5

Groups were compared using Student’s *t*-tests and Chi-square analysis. Values represent the group percentage, or mean ± SEM.

* Significant differences between groups p<0.05, corrected for multiple comparisons.

†ASI – Addiction Severity Index composite scores range from 0 to 1.0, with higher scores indicating greater problem severity.

### Drug/alcohol use

As shown in Table 5, Aboriginal women were more likely to be current cigarette smokers [χ^2^(1)=17.16, p=0.0001], to report more days of cannabis use in the past month [t(165)=3.94, p=0.0001], and more lifetime years of cannabis use [7.9 vs. 3.1; t(164)=3.90, p=0.0001]. Aboriginal women also spent more money in the past month on drugs and alcohol (Table 5), although only differences in alcohol spending were significant [t(162)=2.50, p=0.01]. Significantly more Aboriginal women reported past treatment for a drug or alcohol problem [χ^2^(1)=5.89, p=0.015]; however, it is notable that there were no significant differences in rates of current substance abuse problems.

**Table 5.** Drug/alcohol use stratified by ethnocultural background (n=172).

	Non-Aboriginal (n=89)	Aboriginal (n=83)
Currently smokes cigarettes*	51.7%	81.7%
**Current substance abuse problem**	49.4%	57.8%
**Number of days used in past 30 days (**± **SEM)**		
Alcohol (any use)	2.4 ± 0.6	4.4 ± 0.9
Cannabis*	1.1 ± 0.5	6.0 ± 1.2
Cocaine	1.1 ± 0.5	1.6 ± 0.7
**Amount of money spent on alcohol (past 30 days) (**± SEM)*	$25.63 ± $9.52	$114.10 ± $35.97
**Amount of money spent on drugs (past 30 days) (**± **SEM)**	$38.23 ± $16.72	$92.44 ± $30.21
**Mean ASI Alcohol Composite Severity Score (± SEM)†**	0.10 ± 0.02	0.17 ± 0.2
**Mean ASI Drug Composite Severity Score (± SEM)†**	0.06 ± 0.01	0.07 ± 0.01
Ever treated for drug/alcohol problem*	29.9%	48.1%

Groups were compared using Student’s t-tests and Chi-square analysis. Values represent the group percentage, or mean ± SEM.

* Significant differences between groups p<0.05, corrected for multiple comparisons.

†ASI – Addiction Severity Index composite scores range from 0 to 1.0, with higher scores indicating greater problem severity, measured over the past 30 days

### Mental health

Both Aboriginal and non-Aboriginal women reported high levels of recent and lifetime psychological distress (Table 6). Non-Aboriginal women reported significantly higher rates of depression during the past 30 days [χ2(1)=6.94, p=0.008], and significantly more days of psychological problems in the past month [t(165)=−3.73, p<0.0001]. Non-Aboriginal women were also significantly more likely to be prescribed medication for psychological or emotional reasons in the past month [χ^2^(1)=12.03, p=0.001] and in their lifetime [χ^2^(1)=5.54, p=0.019]. Lifetime rates of depression, anxiety and suicide attempts were high, with no difference between groups (Table 5). Overall ASI psychological composite severity scores were significantly lower among Aboriginal women compared with non-Aboriginal women [t(163)=−2.57, p=0.011].

**Table 6.** Psychological distress stratified by ethnocultural background (n=172).

	Non-Aboriginal (n=89)	Aboriginal (n=83)
**Experienced in past 30 days**		
Depression *	51.7%	31.7%
**Anxiety**	58.6%	43.9%
Prescribed psychiatric medication *	46.0%	20.7%
# Days experienced psychological problems in past month (± SEM)*	16.6 ± 1.4	9.3 ± 1.3
**Mean Beck Depression Inventory score in past week (± SEM)**	19.8 ± 1.5	17.5 ± 1.6
Mean ASI Psychological Composite Severity Score (± SEM)†*	0.39 ± 0.03	0.28 ± 0.03
**Lifetime**		
Ever prescribed psychiatric medication *	66.7%	48.8%
**Ever sought help for a psychological problem**	75.9%	62.2%
**Experienced in lifetime**		
**Depression**	75.9%	78.0%
**Anxiety**	77.0%	73.2%
**Attempted suicide**	50.6%	59.8%

Groups were compared using Student’s *t*-tests and Chi-square analysis. Values represent the group percentage, or mean ± SEM.

* Significant differences between groups p<0.05, corrected for multiple comparisons.

†ASI – Addiction Severity Index composite scores range from 0 to 1.0, with higher scores indicating greater problem severity, measured over the past 30 days.

## Discussion

The majority of the sample were single, socioeconomically disadvantaged women. Low SES was evident for both Aboriginal and non-Aboriginal women given the high rates of unemployment, low educational attainment, reliance on welfare and unstable housing. ASI employment severity ratings were extremely high for both groups, and all women in this study were well under Canadian low-income standards, indicating financial hardship [22, 39].

Women accessing shelter services often report financial difficulty and high rates of lifetime abuse [28]. For example, the 2014 Transition Home Survey indicated that the majority of women accessing shelter services were fleeing various types of abuse (66% emotional abuse, 50% physical abuse, 38% financial abuse, 21% sexual abuse), or were seeking to protect their children from experiencing or witnessing abuse [40]. In this sample lifetime physical abuse was pervasive, although rates were significantly higher among Aboriginal women. Aboriginal women were also significantly more likely to report being the victim of a crime or assault within the past year. Group differences in the prevalence of physical abuse are largely consistent with past reports [18,22], and women accessing shelter services have shown similar, high rates of physical abuse [40,41].

In addition to high rates of physical abuse, childhood sexual abuse (CSA) was also widespread. Although the rates of CSA did not significantly vary across groups, Aboriginal women reported significantly greater severity of CSA compared with non-Aboriginal women. Aboriginal women were also more likely to report having sex with an adult before the age of 14. Aboriginal women were significantly more likely to report one or both parents having a drug/alcohol problem, and troubled family relationships were apparent.

Aboriginal women were also more likely to have had a teenage pregnancy. Teenage pregnancy has been identified as a major health and social issue for both Aboriginals [42–44] and non-Aboriginals [45–47], and is a well-established risk factor for physical abuse [48–51]. The higher rate of teenage pregnancy among Aboriginal women may partly explain the significantly higher rates of physical abuse. Consistent with the findings of this study, teenage pregnancy is also linked with lower educational attainment, lower rates of employment, greater likelihood of achieving or maintaining a low SES, higher rates of maltreatment and an increased risk for poor health among both the mother and child [52–55].

Very few differences were observed between Aboriginal and non-Aboriginal women in terms of physical health, although health issues were common. The majority of the sample reported pain-related issues in the past year, high rates of chronic medical problems and frequent past-month physical problems. These rates of physical illness have been echoed in another recent study of women accessing shelter services, which indicated high rates of cardiovascular issues and sexually transmitted infections [56]. It is important to note that in the current study both ethnocultural groups reported very similar rates of economic disadvantage, and they were of similar SES. Past surveys of the general population found disparities in health between Aboriginal and non-Aboriginal women [3]; however, there were no controls for confounds related to SES. In the present study, irrespective of Aboriginal heritage, women with low SES appear to frequently report poor physical health.

When examining mental health, both Aboriginal and non-Aboriginal women reported moderate levels of substance use, and high levels of lifetime psychological distress. Nearly three-quarters had serious anxiety in their lifetimes, and more than half of non-Aboriginal and Aboriginal women reported a previous suicide attempt. The rate of depression in this sample was considerably higher than the Canadian general population: 11.3% as reported in the 2012 Canadian Community Health Survey – Mental Health (CCHS-MH) [1]. Similar findings have been previously demonstrated when comparing low SES women residing in shelters with a community sample [57]. Although several studies have suggested mental health is a serious issue among Aboriginal communities [30,31,58,59], in this study non-Aboriginal women reported significantly worse psychological health in the past month when compared with Aboriginal women of similar SES. They had higher rates of past-month depression, were more likely to be prescribed a medication for a psychological reason, and reported more days of psychological problems. While it is not possible to determine the source of these differences, it is possible to speculate that there may be significant cultural differences in the expression and reporting of distress (substance use vs. depression), combined with the reported differences in treatment seeking. Alternatively, these differences may reflect a greater sensitivity to developing psychological distress following negative events. Engaging in traditional Aboriginal activities has been linked to better mental health, lower rates of suicide and attenuated drug and alcohol abuse [60–62], and it is possible to speculate that aspects of Aboriginal heritage may have provided some resilience against depression in this sample.

While this study provides insight into the mental health of help-seeking urban women, there are several limitations that should be addressed. Most notably, this study specifically sampled an economically disadvantaged population, and it is unknown whether these results can be generalised to rural communities, or to women from different socioeconomic strata. Despite these limitations the findings indicate that there are serious challenges facing both non-Aboriginal and Aboriginal women in the urban milieu. Adverse childhood events including child neglect, comorbid substance abuse and psychiatric problems in parents, witnessing violence in the home and experiencing physical and sexual abuse are a significant part of their histories. This is especially true for Aboriginal women, given that they reported higher severity of CSA.

Future research should explore the effects of individual resources (e.g. social support, family relations) and cultural beliefs on women’s ability to cope with the stress of living with adverse events, particularly among low SES women with children. Aboriginals migrating to the urban environment may be at particular long-term risk for unstable housing and economic disadvantage given their higher number of dependents, higher amounts of money spent on alcohol and cigarettes, higher rates of experiencing violence/crime and their lack of social support (fewer family members in the urban milieu compared with non-Aboriginals). Longitudinal follow-up studies would be required to determine longer-term outcomes for this urban help-seeking population.
